# An analysis of the trickle-down effect of supervisor knowledge hiding on subordinate knowledge hiding based on displaced aggression theory

**DOI:** 10.3389/fpsyg.2022.1002277

**Published:** 2022-09-21

**Authors:** Yanzhao Tang, Hong-Ming Zhu, Xingcheng Du

**Affiliations:** School of Management, Xiamen University, Xiamen, China

**Keywords:** knowledge hiding, revenge motivation, traditionality, trickle-down effect, displaced aggression

## Abstract

The harm of horizontal knowledge hiding behavior (colleague–colleague) to individuals and organizations has been discussed and confirmed by many studies. The negative consequences of top-down (supervisor–subordinate) knowledge hiding have now emerged as a new focus of research. This study aims to enrich the understanding of the consequences of supervisor knowledge hiding by exploring its trickle-down effect and mechanism. Based on the displaced aggression theory in psychology, this paper analyses and examines the cognitive psychological process and mechanism informing employee knowledge hiding from colleagues when faced with their supervisor’s malicious knowledge hiding behavior. Using a three-stage time-lag questionnaire survey strategy, we collect 233 valid samples of full-time employees from representative provinces and cities in China, covering multiple industries. The following findings are observed: (1) Supervisor knowledge hiding from subordinates (SKHS) positively affects subordinate knowledge hiding from colleagues (SKHC); (2) Revenge motivation plays a mediating role; (3) Traditionality weakens the influence of supervisor knowledge hiding on a subordinates’ revenge motivation. This study confirms the trickle-down effects of supervisor knowledge hiding behavior, extends research on the consequences of top-down knowledge hiding and its mechanism and provides new insights for organizational practice.

## Introduction

Knowledge hiding has emerged as a significant issue and popular research topic in recent years, and the potential consequences of knowledge hiding behaviors have been extensively studied. Connelly et al. (2012) defined knowledge hiding as “an intentional attempt by an individual to withhold or conceal knowledge that has been requested by another person.” This behavior violates the ethical values of organizations and hinders knowledge exchange among employees and knowledge dissemination within an organization. It inhibits positive behaviors, such as innovation and creation (Černe et al., 2017; Arain et al., 2020c; Duan et al., 2022), and triggers deviance and counterproductive behavior (Singh, 2019), causing severe damage to both organizations and individuals (Bavik et al., 2018).

The majority of research on the detrimental impacts of knowledge hiding has focused on horizontal knowledge hiding or employees’ knowledge hiding from coworkers in the same organizational hierarchy. While top-down knowledge hiding behavior, or knowledge hiding by supervisors from subordinates, has received less research attention (Arain et al., 2020c,^2022^), the behaviors are also prevalent and could be more harmful (Connelly and Zweig, 2015). Supervisors may purposefully mislead or conceal information to avoid providing knowledge assistance to subordinates, which may be motivated by a variety of factors, including the need to protect one’s own unique knowledge, avoid losing face, or maintain one’s position of authority (Butt, 2020). These behaviors can harm employee self-esteem, creativity, and work performance (Arain et al., 2020c; Abdelmotaleb et al., 2022).

A small number of existing empirical studies on top-down knowledge hiding have validated the negative impact of supervisor knowledge hiding behaviors on individuals and organizations (Offergelt et al., 2019; Arain et al., 2020a,b, c, 2022; Abdelmotaleb et al., 2022). For example, Arain et al. (2020a) found that identifying supervisor knowledge hiding behavior could weaken subordinates’ perception of trust and reduce organizational citizenship behavior. Offergelt et al. (2019) found that employees’ perception of knowledge hidden by their supervisors was positively correlated with their knowledge hiding and turnover intentions. Abdelmotaleb et al. (2022) found that leaders’ knowledge-hiding behavior negatively affected employees’ organizational identity through the mediating effect of self-interested climate perception.

The few studies on top-down knowledge hiding behavior are primarily concerned with how supervisor knowledge hiding affects outcomes like attitudes, behaviors, and performance at the individual and organizational levels, while the potential trickle-down effects of supervisor knowledge hiding itself as well as its mechanisms are largely ignored. Given that the negative effects of employee knowledge hiding on both individuals and organizations have been discussed and supported by a large number of studies (Arain et al., 2020c), if the trickle-down effect of supervisor knowledge hiding (Mawritz et al., 2012) does exist, it will be more detrimental to the organization than horizontal knowledge hiding (Arain et al., 2020c). Although two studies (Offergelt et al., 2019; Arain et al., 2022) have explored the effects of supervisor knowledge hiding on employee knowledge hiding, the specific path and mechanism remain unclear. In light of this, we focus on the effects of supervisor knowledge hiding on subordinate knowledge hiding in this work.

The following arguments are presented in this research: (1) Supervisor knowledge hiding positively affects subordinate knowledge hiding; (2) The revenge motive plays a mediating role; (3) Traditionality weakens the influence of supervisor knowledge hiding on a subordinate’s revenge motive. This paper defines supervisor knowledge hiding from subordinates as a deliberate and destructive implicit infringement conduct in which the subordinate perceives that the behavior is deliberately carried out by the supervisor and causes damage to themselves (Baron, 2005). According to the theory of displaced aggression, people who have experienced unfair treatment, such as playing dumb or evasive knowledge hiding from their supervisor, will turn to other innocent objects for displaced aggression if they are unable to directly retaliate against the aggressor (Dollard et al., 1939). A centralism culture of subordination to authority may increase the phenomenon of displaced aggression. Due to a supervisor’s greater rank and authority, a subordinate who encounters their knowledge hiding may not take direct action against the supervisor but instead may extend the bad behavior to others who are more susceptible to attack (Dollard et al., 1939). We test our hypothesis using unique survey data from China, a classic centralist environment that perfectly suits the demands of this study.

This paper provides the following theoretical contributions: First, we find that supervisor knowledge hiding can “trickle-down” and lead to subordinate knowledge hiding, establishing the existence of the trickle-down effect in the field of knowledge hiding. In addition, we respond to the appeal by Connelly and Zweig (2015) for research into top-down knowledge hiding and add to the research on the consequences of supervisor knowledge hiding; Second, drawing on the displaced aggression theory, this research investigates the cognitive psychological process that explains why a victimized individual engages in knowledge hiding from coworkers when perceiving knowledge hiding by a supervisor, thereby enhancing the understanding of the trickle-down mechanism of supervisor knowledge hiding. We find that supervisor knowledge hiding is an intentional infringement, which will not only cause the subordinates to accept it silently and react negatively but also trigger more severe reactions and exacerbate harm to an organization. Third, this study demonstrates that traditionality can reduce the reactions of subordinates to supervisor knowledge hiding with malicious intent. This finding expands the body of knowledge on traditionality’s role in dampening the influence of negative leader behavior and contributes to the research of factors that strengthen or weaken the trickle-down effect.

## Theory and hypotheses development

### Knowledge hiding

Knowledge hiding originated from a focus on knowledge sharing. Research on knowledge-sharing behavior found that when employees possess valuable knowledge, they are often reluctant to share it with their coworkers in favor of keeping it to themselves (Argote and Ingram, 2000). In addition, since knowledge is an exclusive resource, the organization cannot force its employees to share it. Due to the existence of “knowledge-sharing hostility,” individuals have a strong incentive to avoid sharing knowledge, which results in knowledge hoarding and rejecting behavior in an organization (Husted et al., 2012).

Connelly et al. (2012) first explored knowledge hiding behavior within organizations through experience sampling methodology. They established that knowledge hiding existed in organizations, clarified its concept and connotations, and introduced it as an individual-level concept in the research of organizational behavior. Specifically, knowledge hiding is defined as “an intentional attempt by an individual to withhold or conceal knowledge that has been requested by another person.” The knowledge in the above definition includes opinions, ideas, advice, and information, as well as the unique experience and specialized knowledge relevant to the accomplishment of organizational activity. There are various ways that knowledge hiding behavior might appear in corporate settings. Connelly et al. (2012) distinguished three types of knowledge hiding. The first is evasive hiding, in which knowledge hiders intentionally mislead knowledge seekers by providing false knowledge and information or by using delaying tactics to keep their knowledge hidden. The second is playing dumb, in which knowledge hiders purposefully demonstrate that they do not comprehend the demands for knowledge and the issues facing knowledge seekers, or they pretend to lack the requested knowledge. The third is rationalized hiding, in which knowledge hiders don’t provide knowledge to others for an objective reason, like the knowledge requested is confidential. It can be seen that evasive hiding and playing dumb are obviously malicious and deceptive, whereby the hiders have no actual willingness to provide knowledge assistance. Knowledge hiding is subtly presented and cannot be judged by the subordinate in a short period of time (Connelly et al., 2012). However, due to the long-term relationship between supervisors and subordinates, subordinates have basic judgments about their supervisor’s work ability and primary work arrangements, and thus have an overall perception of the supervisor’s capacity and availability to fulfill their knowledge needs (Arain et al., 2020a,b; Tremblay et al., 2022). Therefore, the malicious knowledge hiding behavior of supervisors can be perceived by subordinates.

The negative effect of knowledge hiding has drawn a lot of attention from academics as a behavior that harms both organizations and individuals. Knowledge hiding behavior limits access to knowledge and information for the knowledge seeker and has a detrimental effect on personal creativity and performance (Černe et al., 2014). When the knowledge seeker feels violated by the knowledge hider, they may start to implement their own hiding of knowledge from the knowledge hider, which will reduce their assistance and support. A vicious spiral is created for knowledge hiders as a result of their behavior, which also harms their own performance, creativity, and reputation (Černe et al., 2014; Evans et al., 2015). At a team level, knowledge hiding destroys the trust between team members and stifles knowledge interchange, which is unfavorable to team members’ skill development and knowledge augmentation (Černe et al., 2014) and has a detrimental effect on innovative behavior and output (Baer et al., 2015). As for the organization, employees’ knowledge hiding hinders the transfer of knowledge, resulting in a decline in the efficiency of information exchange and communication within an organization, which will reduce the firm’s capacity for innovation and advancement.

Knowledge hiding behavior among employees of the same level has been extensively explored. However, this behavior does not exist among employees only, and the knowledge hiding of supervisors from subordinates deserves more attention, especially its negative impact on organizations and individuals (Connelly and Zweig, 2015; Connelly et al., 2019). The most recent relevant research has found that a supervisor’s knowledge hiding has a negative impact on employee trust, self-efficacy, organizational identity, innovative behavior, organizational citizenship behavior, personal performance (Arain et al., 2020a,b, 2022; Abdelmotaleb et al., 2022), etc., and a positive effect on moral disengagement, silence to superiors, team interpersonal deviance, turnover intention (Offergelt et al., 2019; Arain et al., 2020c,^2022^), etc. However, there is still much to learn about the harm caused by supervisor knowledge hiding. This paper investigates the substantial trickle-down effect of supervisor knowledge hiding and explores its mechanism of harm to organizations.

### Trickle-down effect

Trickle-down effects refer to “the flow of perceptions, feelings, attitudes, and behaviors down the organizational hierarchy” (Wo et al., 2018), in which “perceptions, attitudes, or behavior of one person in the organization (usually a supervisor) trickle-down through the organizational hierarchy and influence perceptions, attitudes, or behavior of another person (usually a subordinate)” (Wo et al., 2015). Masterson (2001) was the first to define and analyze trickle-down effects in organizations, who examined a trickle-down model of organizational justice and found that employees’ perceptions of fairness from organization can affect their sentiments toward the organization, subsequently influencing how they behave toward customers.

Since then, the trickle-down effect has been observed in numerous areas, such as perceptions of justice (Hoobler and Hu, 2013), abusive supervision (Mawritz et al., 2012), breaches and violations of psychological contracts (Bordia et al., 2010), calling orientation (Xie et al., 2019), work engagement (Lu et al., 2018), empowering leadership (Byun et al., 2020), servant leadership (Stollberger et al., 2019), and ethical leadership (Babalola et al., 2019).

Displaced aggression theory is used to explain the mechanism of trickle-down effect from an affective perspective. Another two commonly used theories are social learning theory and social exchange theory (Wo et al., 2015). According to the displaced aggression theory, people will feel anger and frustration due to the aggressive behavior of others, and if they are unable to immediately respond to the aggressor, they will turn their wrath and frustration toward others in an effort to divert their negative feelings (Dollard et al., 1939). The current study focuses on the trickle-down effect and mechanism of supervisor knowledge hiding behavior based on displaced aggression theory.

### Supervisor knowledge hiding from subordinates and subordinate knowledge hiding from colleagues

The supervisor serves as both a role model and a work coach in a workplace and is a valuable resource for employees to obtain organizational feedback, which will have a significant effect on employee behavior (Ilgen et al., 1979; Mawritz et al., 2012). Supervisor and subordinate behavior frequently align with the social exchange and reciprocity norm (Blau, 1964). However, the aforementioned justification does not adequately capture the deliberate and invasive nature of supervisor knowledge hiding behavior. This study defines supervisor knowledge hiding as an intentional and damaging implicit infringing behavior in which a subordinate believes that the supervisor’s actions are deliberate and harm them as a result (Baron, 2005). The key elements of malicious knowledge-hiding activity that supervisors engage in with subordinates are immorality, unfairness, and harmfulness. The subordinates will blame the supervisor for interpersonal unfairness and intentional infringement they experience and form hostile attributions.

Retaliation has emerged as a crucial perspective in related research on workplace aggression, deviance, and unethical behavior (Mitchell and Ambrose, 2007). Retaliation is a behavior tendency or mode of action in which people who have experienced harm or are irritated wish to hold the guilty accountable for their wrongdoings or misdeeds (Skarlicki et al., 1999). Subordinates who experience knowledge hiding by their supervisors will feel frustrated, helpless, and alienated and will lose their identification and perception of fairness and justice in the organization (Butt, 2020), resulting in negative emotions such as anger. According to the cognitive process model of “frustration-aggression,” when hostile attribution and negative energy gradually build up, subordinates frequently engage in destructive acts to respond or vent (Faldetta, 2021). Thus, retaliation is a proactive tactic used to combat their supervisor’s knowledge-hiding activity (Bies and Tripp, 1996; Hershcovis et al., 2007).

According to the displaced aggression theory, as there is a chance of retribution and conflict escalation in retaliation against their supervisors directly, subordinates who are the victims of knowledge hiding by their supervisors will turn to their coworkers with displaced aggression. As supervisors are high-ranking, subordinates will make an effort to avoid “tit for tat” against their supervisors directly (Mackey et al., 2015), especially within the cultural background of “subordination to authority.” As a result, subordinates will seek the more convenient objects of their colleagues to transfer their negative state by engaging in harmful or immoral behaviors, such as their own knowledge hiding, in an attempt to balance out the psychological effects and perceptions of professional setback. This paper proposes the following assumptions:

Hypothesis 1: SKHS has a positive effect on SKHC.

In order to fully understand the relationship between supervisor knowledge hiding and subordinate knowledge hiding, it is necessary to further investigate the underlying motivational mechanism of this relationship. For this purpose, we again employ displaced aggression theory and explore revenge motivation as a mediator in the relationship between supervisor and subordinate knowledge hiding.

### Mediation of subordinate’s revenge motivation between supervisor knowledge hiding and subordinate knowledge hiding

#### Supervisor knowledge hiding from subordinates and subordinate revenge motivation

Revenge motivation is a behavioral intention in which individuals seek to hurt others by means of attack or infringement when they encounter damage or setbacks in interpersonal communication so as to vent their emotions and dissatisfaction (Bies and Tripp, 1996; Aquino et al., 2001). According to relevant research on human evolution, revenge motivation has evolved into a prevalent human trait via natural selection (McCullough, 2008), and its purpose is to punish and avenge experienced aggression rather than merely to stop it. Evidently, after being violated or treated unfairly by others, retaliation motivation and even reprisal behavior are frequently unavoidable (Bradfield, 1999). The genesis process of the revenge motive is explained by the cognitive process model put forth by Beugré (2005). According to Beugré (2005), people are not naturally motivated to act negatively after encountering terrible occurrences; rather, it is the outcome of a number of cognitive phases. In addition, the fulfillment of individual resource demands plays a significant role in the generation of retaliation motivation. According to the conservation of resources theory, individuals have the intrinsic motivation to acquire new resources, maintain existing resources, and prevent the rapid depletion of their own resources (Hobfoll, 1989). When external conditions are favorable, employees will actively manage their relationships with coworkers and put in more effort at work to acquire new resources; when external situations are unfavorable, employees will use their available resources to deal with them. If such resource consumption surpasses the level an employee can tolerate, it will result in significant psychological pressure and alienated workplace behavior (Hobfoll, 1989, 2011).

Supervisors are legitimate sources of organizational support and knowledge for subordinates, and their malicious knowledge hiding behavior unfairly infringes on the legal rights and interests of the subordinates (Arain et al., 2020b) and breaks accepted norms among organizational members (Bies and Tripp, 2005). While this aggressive behavior reduces the resources or support received by subordinates, it also damages their rights and depletes extra personal resources, which undermines their autonomy and inhibits their initiative to actively reach their potential (Butt, 2020). In addition, because of the supervisors’ malicious knowledge hiding behavior, subordinates will lack the necessary knowledge and information to solve problems and will be unable to approach work assignments creatively (Butt, 2020), weakening their job competency. Coworkers will also reduce expectations for the employee because they believe that they lack the necessary skills and expertise to carry out their responsibilities (Butt, 2020). Thus, the autonomy, competence, and sense of belonging of the subordinates will all be harmed as a result of the supervisors’ knowledge concealment, and as a result, they will feel violated and unfairly treated by the supervisor. The perceived aggression and injustice of subordinates will further enhance their revenge motivation to even out the relationship (Skarlicki and Folger, 1997; Jones, 2009).

Hypothesis 2: SKHS has a positive effect on the subordinate’s revenge motive.

#### Subordinate revenge motive and subordinate knowledge hiding from colleagues

Motivation serves as a link between an individual’s conduct and the external environment or stimuli they are exposed to. An individual’s behavior is typically driven by their motivation. Not only does motivation cause behavior to occur, it can also have an impact on how long it lasts. It also plays an important role in adjusting behavior objects and behavior styles (Deci and Ryan, 1985). In addition to expressing dissatisfaction with the malevolent actions of their superiors, subordinates will demonstrate a willingness to alter their behavior in reaction to the unjust treatment (Adams, 1965).

Drawing on the theory of displaced aggression, we believe that instead of retaliating against their superiors directly, subordinates may choose to implement their retaliation in more covert ways, such as knowledge hiding from coworkers, primarily for the following two reasons: first, subordinates interact and communicate with their coworkers more frequently than they do with their supervisors, which provides more opportunities for subordinates to implement deviant behaviors directed toward colleagues rather than toward supervisors (Wei and Si, 2013); Second, because the supervisor is in a position of greater authority and status than the subordinates, they are more likely to take retaliatory action. Therefore, taking direct action against a supervisor has greater risks and potential costs. Subordinates can believe that their coworkers are not in a position to penalize them for their inappropriate behavior compared to a supervisor. As a result, subordinates may pick coworkers as substitutes to transfer or release negative feelings, such as anger, frustration, and resource depletion (Wang and Noe, 2010). Because it is deceptive, malicious knowledge hiding behavior toward colleagues is blatantly aggressive and damaging and is difficult to spot. This displaced aggression not only achieves the purpose of revenge but provides a vent for negative feelings and will not trigger counter-retaliation and punishment.

To summarize, the malicious knowledge hiding behavior of the supervisor may induce revenge motivation in the subordinate. Under rational consideration, the subordinate will implement malicious knowledge hiding behavior to the colleagues.

Hypothesis 3: The subordinate revenge motive has a positive effect on SKHC.

#### The mediating role of subordinate’s revenge motive

Based on the above analysis, a supervisor’s malicious knowledge hiding leads to a failure to meet the resource needs of subordinates, which damages their autonomy, competence, and sense of belonging. The subordinates will carry out hostile attribution after determining that the source of their frustration and victimization is the supervisor (Aquino et al., 2001). In order to transfer the negative states generated, as well as to compensate and balance the exchange relationship, the subordinates will have a desire for revenge (Bies and Moag, 1986; Bies and Tripp, 1998). To directly retaliate against the supervisor involves danger and difficulties due to the supervisor’s authority and status. Thus, according to the rational cognitive process, the subordinate will transfer the knowledge hiding behavior they experience to a target that is simpler to attack, i.e., implement malicious knowledge hiding to coworkers as payback (Dollard et al., 1939).

Hypothesis 4: Subordinate revenge motivation mediates the relationship between SKHS and SKHC.

### Moderating effects of subordinate traditionality

As mentioned above, supervisor knowledge hiding behavior triggers the “frustration-aggression” cognitive process of the subordinate, in which subordinate revenge motivation is generated, and they respond with negative attitudes, emotions, and behavior. However, this cognitive process is not always present and is affected by personal traits (Deci and Ryan, 1985). Subordinates will interpret and respond differently to the impact and damage caused by external irritant events depending on their traits. The trickle-down effect of knowledge hiding studied in this research occurs at different levels of organizational hierarchy. We believe that employees’ subjective identification with hierarchical relationships may alter the trickle-down effect. Specifically, we analyze the impact of subordinate traditionality on supervisor knowledge hiding and the negative coping mechanisms of subordinates.

Yang (2003) defined individual traditionality as a “typical pattern that is more or less related to motivational, evaluative, attitudinal, and temperamental traits most frequently observed in people in traditional Chinese society, which can still be found in people in contemporary Chinese societies.” This traditionality manifests itself in five aspects, including submission to authority, filial piety and ancestor worship, conservatism and endurance, fatalism and defensiveness, and male dominance (Yang et al., 1989). By concentrating on the dimension of submission to authority, Farh et al. (1997) introduced the construct of traditionality to organizational research. Following their work, Farh et al. (1997) defined traditionality as “the degree to which an individual supports the traditional hierarchical role relationships advised by Confucian social ethics.”

Traditional Confucian values place a strong emphasis on benevolence and forgiveness. When faced with unjust treatment by authority figures, people are not encouraged to blame them because expressing dissatisfaction with supervisors and other authoritative figures goes against traditional beliefs (Liu et al., 2010). In addition, this kind of value orientation posits that fairness and justice will eventually manifest themselves rather than encouraging the balancing of individual rights and interests through retaliation. In the workplace, the allegiance and obedience of subordinates to their superiors in role relationships is the most obvious manifestation of traditionality (Hui et al., 2004). One of the requirements for success in an organization, particularly for high traditionalist employees, is to keep positive interpersonal relationships with their superiors. Subordinates’ dissatisfaction or negative attitudes toward their superiors will damage their interpersonal relationships, thus impeding their ability to execute their jobs effectively and advance their careers within the company (Liu et al., 2010). Prior research has found that traditionality moderates the relationship between justice and organizational citizenship behaviors (Farh et al., 1997), transformational leadership and leader effectiveness (Spreitzer et al., 2005), inclusive leadership and follower’s taking charge (Wang et al., 2020), leader humility and employees’ proactive behavior (Chen et al., 2021), supervisor’s mentoring quality and subordinate’s proactive behavior (Wu et al., 2019). Traditionality can also moderate the relationships among parental support, career decision-making self-efficacy and career adaptability (Guan et al., 2016). Knowledgeable workers who possess negotiating skills in their relationships with supervisors can have different levels of traditionality (Huo et al., 2014). From the recent work examining the moderating role of traditionality in the relationship between inclusive leadership and follower’s taking charge, most of the samples (65.85%) are under 30 years of age and have a master’s degree (Wang et al., 2020). Similarly, the research sample age of career construction and cognitive evaluation under the effect of traditionality in Guan et al. (2016) is a mean of 21.29 years. Thus, we can get the point that the research of traditionality on the younger generation of knowledge employees is applicable.

**FIGURE 1:**
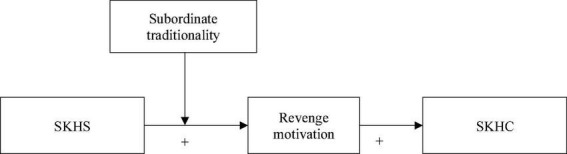
Hypothesized model.

We suggest that subordinate traditionality serves as a boundary condition for the relationship between SKHS and revenge motivation (Figure 1). The higher the subordinate’s traditionality, the less likely it is that supervisor knowledge hiding will incite a desire for retribution. This is due to the following: First, high traditionality subordinates emphasize maintaining a harmonious and intimate relationship with their superiors and anticipate receiving their approval, which is a crucial means of improving their reputation and self-worth (Farh et al., 2007). High traditionality subordinates typically define themselves and assess their status and responsibilities inside the company based on their relationship with superiors (Farh et al., 2007). Therefore, the motivation to retaliate or foster the idea of engaging in deviant behavior is often difficult for them to generate (Liu et al., 2010). Furthermore, because subordinates with high levels of traditionality are more self-disciplined and make self-attributions for the potential negative effects of knowledge hiding (such as inefficiency and lack of competence), they may also believe that a supervisor’s malicious knowledge hiding behavior is reasonable. Instead of blaming the supervisor for the mistreatment they experience, high traditionality subordinates place the blame on themselves (Schilpzand et al., 2016).

Hypothesis 5: Subordinate traditionality moderates the relationship between SKHS and revenge motivation in such a way that the positive relationship is stronger for those with less, rather than more, traditionality.

## Materials and methods

### Sample and procedure

A survey was administered to full-time employees of companies located in seven provinces of China, including Gansu, Shaanxi, Fujian, Beijing, Shanghai, Guangdong, and Sichuan. The surveyed companies were small and medium-sized companies in industries such as manufacturing, service, construction, IT, water conservancy, and environmental industries. The selected survey targets were basic-level technicians whose work demand professional knowledge and skills, especially unique knowledge accumulated through time. We identified such employees based on their work type and position. Since the purpose of this paper is to explore the trickle-down mechanism of the supervisor’s knowledge hiding to the knowledge hiding of the subordinate, the whole psychological process and mechanism of the subordinate from the perception of supervisor knowledge hiding behavior to the implementation of knowledge hiding to colleagues was analyzed. Thus, all the variables in this paper are taken from the employee survey self-reports.

A three-wave time-lag data collection strategy was employed in this work, with an interval of roughly 2 weeks between each wave, in an effort to reduce the potential for common method bias (Podsakoff et al., 2003). We distributed 388 questionnaires in the first wave, of which 383 were completed, and perceived supervisor knowledge hiding and control variables were gathered. The second questionnaire measured revenge motivation and traditionality. Of the 383 respondents, 328 participated in the second wave, and 320 completed the survey properly. The third questionnaire measured knowledge hiding from coworkers, in which 282 respondents participated and 281 completed the survey properly. Questionnaires from the three waves were matched using the last four digits of the respondents’ mobile phone numbers, and invalid questionnaires were excluded. In total, 233 questionnaires were matched and valid, and the effective recovery rate of the questionnaires was 60.1%, yielding a sample size large enough to perform relevant statistical analysis (Arain et al., 2020a).

In the final sample, 45.5% were male, and 54.5% were female. In total, 28.8% were between 21 and 30 years old, 43.3% were from 31 to 40 years old, and the rest were over 40. Of these respondents, 35.2% had bachelor’s degrees, 29.6% had master’s degrees or PhDs, and the remaining had associate’s degrees or less. As for the organizational tenure, 52.4% of the employees had 1–5 years of service in their current enterprises, 21.4% of them had 6–10 years of service, and the remaining employees had more than 10 years. In terms of working hours per week, 41.2% worked 40 h per week, 23.2% worked less than 40 h in a week, and the rest worked more than 40 h in a week.

### Measures

The four main variables of this study, SHKS, revenge motivation, traditionality, and SHKC, all adopted mature measures according to the existing literature. Most of the scales were previously translated and retranslated by senior scholars in relevant fields in China, and the reliability and validity of the post-translation scale in the context of Chinese organizational culture had been verified, providing a solid foundation for this study. People tend to underreport sensitive occurrences like knowledge hiding since it may be seen as socially undesirable (Connelly et al., 2012). The questionnaires were anonymous, and we guaranteed the confidentiality of respondents’ information and responses to prevent falsely disguised responses (McGrath, 2001). All the variables were measured on a Likert scale ranging from 1 (strongly disagree) to 5 (strongly agree).

SKHS was measured using eight items from a scale developed by Connelly et al. (2012), which consisted of four items each for evasive hiding and playing dumb. The four items for rationalized hiding in the original scale were not included because of our focus on deliberate and destructive infringement conduct. Respondents were asked to respond to the following statement, “For a moment, visualize the supervisor you request knowledge from: how do they behave?” A sample item of evasive hiding is “My supervisor agrees with me but never really intends to provide me with the requested information.” A sample item of playing dumb is, “My supervisor says that they do not know, even though they do.” The items were averaged to produce a scale score, and Cronbach’s alpha was 0.890.

Revenge motivation was measured using a four-item scale developed by Jones (2009). A sample item is, “If I were mistreated by a supervisor, it would feel good to get back in some way.” We obtained a Cronbach’s alpha of 0.846.

SKHC was measured using the scale adopted by Jiang et al. (2019), which was an adapted version of the scale developed by Connelly et al. (2012). Consistent with the scale of SKHS, evasive hiding and playing dumb subscales were adopted. A sample item is, “When coworkers requested knowledge from me, I offered them some other information instead of what they really wanted.” The Cronbach’s alpha of this scale in this study was 0.924.

Traditionality was measured using a five-item scale adopted by Farh et al. (1997). A sample item is, “The chief government official is like the head of a household. The citizen should obey his decisions on all state matters.” The Cronbach’s alpha of this scale in this study was 0.859.

Based on previous empirical findings on the consequences of supervisor knowledge hiding (Arain et al., 2020a,^2022^; Abdelmotaleb et al., 2022), the following controls were employed while testing the hypotheses: gender, age, education, organizational tenure, work engagement, and industry.

### Analytical strategy

The hypothesized model was a moderated mediation model in which the mediation effect of revenge motivation between the SKHS and SKHC relationship was further moderated by traditionality, as shown in Figure 1. According to Gefen et al. (2000), hierarchical regression analysis is more appropriate and widely used than structural equation model (SEM) for testing the specific mechanism in a model, particularly for testing a moderating effect. As a result, hierarchical regression analysis was utilized in this study to test the proposed model, and the analysis was carried out in the procedure outlined below: in the first step, we used Amos 23.0 software to create a SEM for confirmatory factor analysis (CFA) in order to verify the critical validity between variables. Then, we performed the Harman’s single factor test, the common latent factor (CLF) test, and other procedures to ensure that common method bias was not a threat to this study. In the second step, we employed a hierarchical regression analysis approach to test the proposed hypothesis using the SPSS 18.0 software. We first examined the main effect of SKHS on SKHC. Next, we tested the mediation effect of revenge motivation using the method recommended by Baron and Kenny (1986). The moderating effect of traditionality was then tested by creating an interaction term between SKHS and traditionality. In the third step, we followed the suggestion of Preacher and Hayes (2008) and uses the bootstrapping procedures to further examine the mediation effect of revenge motivation. We ran PROCESS Model 4 (Hayes, 2013) with 5,000 bootstrap samples. In addition, we tested the moderated mediation effect using bootstrap method following the suggestion of Edwards and Lambert (2007), and ran PROCESS Model 7 (Hayes, 2013) with 5,000 bootstrap samples.

## Analysis and results

### Confirmatory factor analysis

The CFA results are shown in Table 1. According to the criteria outlined by Bentler and Bonett (1980), when the root mean square error of approximation (RMSEA) is below 0.08, the comparative fit index (CFI) is above 0.90, and the Tucker–Lewis index (TLI) is above 0.90, it indicates that the model fits well. The results in Table 1 show that the fit indices of the four-factor model (SKHS, revenge motivation, traditionality, and SKHC) meet the accepted benchmark (χ^2^/df = 2.04, RMSEA = 0.07, CFI = 0.91, TLI = 0.90). In addition, the fit indices of the four-factor model are obviously better than that of other alternative models. This demonstrates the strong discriminant and divergent validity of the four key variables used to gauge the study’s outcomes.

**TABLE 1:** Results of the CFA of the measures of the variables used in the study.

Model	χ^2^/df	RMSEA	CFI	TLI
Four-factor model	2.04	0.07	0.91	0.90
Three-factor model a	3.42	0.09	0.79	0.77
Three-factor model b	3.74	0.11	0.77	0.74
Two-factor model c	5.06	0.13	0.65	0.62
One-factor model	8.01	0.17	0.39	0.34
Threshold value	<3.00	≤0.08	≥0.90	≥0.90

*N* = 233. χ^2^/df, normed chi-square; CFI, comparative fit index, RMSEA, root mean square error of approximation; TLI, Tucker–Lewis index; SKHS, supervisor’s knowledge hiding from subordinates; SKHC, subordinate’s knowledge hiding from colleagues. A revenge motivation and SKHC were combined into one factor; b SKHS and traditionality were combined into one factor; c SKHS and traditionality were combined into one factor, and revenge motivation and SKHC were combined into one factor.

### Common method bias

The risk of common method bias increases when the measurements of all research variables originate from a single source (Podsakoff et al., 2003). We conducted the following procedures adopted by Abdelmotaleb et al. (2022) to address the issue of common method bias. First, time-lag research design was used, and the variables were measured at three time points. Second, the common method bias was examined using Harman’s single factor test. According to the findings of an exploratory factor analysis performed using the SPSS 18.0 software program, the largest factor in this study only accounted for 21.93% of the total variance, which was under the critical threshold of 50%. Thus, there was no indication of common method bias in Harman’s test. In addition, CFA results in Table 1 show that the fit indices of the one-factor model are the worst (χ^2^/df = 8.01, RMSEA = 0.17, CFI = 0.39, TLI = 0.34), demonstrating that the common method bias is not substantial. Furthermore, considering the limits of Harman’s single factor test, this study conducted a CLF test. We added a CLF to the four-factor model, which was connected to all the indicators of the four factors, and CFA was performed. The CFA estimates of the model with a CLF (χ^2^/df = 2.03, RMSEA = 0.07, CFI = 0.91, TLI = 0.90) only had a slight difference from those of the four-factor model and were below the threshold value of 0.20 (Arain et al., 2020b). Consequently, the CLF test revealed no evidence of common method bias. Based on the above analysis, the common method bias did not significantly threaten the validity of the study.

### Descriptive statistics

This study employed SPSS 18.0 software for data processing. Table 2 reports the mean (Mean), standard deviation (SD), and Pearson correlation coefficient of each variable in this study. According to the data analysis results, there is a positive correlation between the SKHS and the revenge motivation of subordinates (*r* = 0.20, *p* < 0.01), as well as a positive correlation between the SKHS and SKHC (*r* = 0.19, *p* < 0.01) and a positive correlation between subordinates’ revenge motivation and SKHC (*r* = 0.19, *p* < 0.05). The results indicate that the correlation of variables is in line with the hypothesis, which serves as the foundation for subsequent regression analysis.

**TABLE 2:** Means, standard deviations, and inter-correlations.

	1	2	3	4	5	6	7	8	9	10
(1) Gender	−									
(2) Age	–0.04	−								
(3) Education	0.02	−0.35**	−							
(4) Organizational tenure	–0.03	0.66**	−0.40**	−						
(5) Work engagement	–0.04	–0.05	0.08	–0.02	−					
(6) Industry	0.06	0.01	0.16*	0.15*	0.15*	−				
(7) SKHS	–0.05	0.08	−0.26**	0.06	–0.05	−0.16*	−			
(8) Revenge motivation	0.07	0.11	0.10	0.01	–0.03	–0.03	0.20**	−		
(9) Traditionality	–0.05	0.11	−0.27**	0.08	−0.15*	–0.09	0.23**	–0.10	−	
(10) SKHC	–0.02	0.04	–0.08	–0.10	−0.17*	−0.19**	0.19**	0.19*	0.14*	−
Mean	1.55	35.89	2.79	8.00	41.82	2.36	3.01	2.48	3.14	2.59
SD	0.50	8.47	1.03	7.54	7.34	1.23	1.00	0.95	0.96	1.08

*N* = 233. SKHS, supervisor’s knowledge hiding from subordinates; SKHC, subordinate’s knowledge hiding from colleagues. **p < 0.01, *p < 0.05.

### Hypotheses testing

The hierarchical regression analysis results are presented in Table 3. According to the results of Model 2, the SKHS significantly positively affects the SKHC (M2, β = 0.17, *p* < 0.05); thus, Hypothesis 1 is supported.

**TABLE 3:** The results of hierarchical regression modeling.

Variables	SKHC				Revenge motivation			
	M1	M2	M3	M4	M5	M6	M7	M8
**Control variables**								
Gender	–0.04	–0.02	–0.07	–0.05	0.14	0.15	0.14	0.11
Age	0.02	0.02	0.01	0.01	0.02*	0.02*	0.02*	0.02*
Education	–0.10	–0.05	–0.13	–0.09	0.14*	0.20**	0.17*	0.14*
Organizational tenure	−0.03*	−0.03*	−0.03*	−0.03*	–0.01	0.00	–0.01	–0.01
Work engagement	−0.02*	−0.02*	−0.02*	−0.02*	0.00	0.00	0.00	0.00
Industry	–0.10	–0.09	–0.09	–0.08	–0.04	–0.02	–0.02	–0.01
**Independent variable**								
SKHS		0.17*		0.13		0.23**	0.25**	0.22**
**Mediator variable**								
Revenge motivation			0.21**	0.18*				
**Moderator variable**								
Traditionality							−0.13*	−0.14*
**Interaction**								
SKHS × traditionality								−0.13*
R^2^	0.08	0.10	0.12	0.13	0.04	0.10	0.11	0.13
ΔR2	0.06	0.08	0.09	0.10	0.02	0.07	0.08	0.10
ΔF	3.35**	3.76**	4.18**	4.10**	1.72	3.41**	3.53**	3.78**

*N* = 233. SKHS, supervisor’s knowledge hiding from subordinates; SKHC, subordinate’s knowledge hiding from colleagues. **p < 0.01, *p < 0.05.

The method suggested by Baron and Kenny (1986) has been widely used for testing the mediation effect and was employed in this work. According to the regression results of M6, SKHS significantly positively affects the subordinate’s revenge motivation (M6, β = 0.23, *p* < 0.01); thus, Hypothesis 2 is confirmed. As shown by the regression results of M3, subordinates’ revenge motivation has a significant positive effect on SKHC (M3, β = 0.21, *p* < 0.01), and Hypothesis 3 is supported. M4 incorporates the mediator variable of revenge motivation based on M2. The results of M4 show that the positive influence of SKHS on SKHC is weakened (M4, β = 0.13, *p* = 0.07), and the subordinate’s revenge motivation still has a significant positive effect on the SKHC (M4, β = 0.18, *p* < 0.05). Therefore, Hypothesis 4 is supported, which indicates that subordinates’ revenge motivation plays a partial mediating role between the SKHS and SKHC.

**FIGURE 2:**
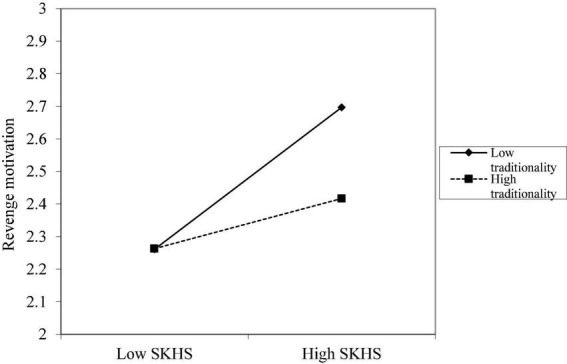
Moderation effect of different levels of traditionality on the relationship between SKHS and subordinate revenge motivation.

Next, we examine the moderating effects of subordinate traditionality. According to the results of model M8 in Table 3, the interaction between SKHS and subordinate traditionality has a significant negative effect on the subordinate’s revenge motivation (M8, β = −0.13, *p* < 0.05), indicating that the subordinate’s traditionality trait will weaken the effect of SKHS on the subordinate’s revenge motivation. Therefore, Hypothesis 5 is supported. Figure 2 depicts the difference in the impact of SKHS on subordinates’ revenge motivation at different levels of traditionality based on one standard deviation above the mean and one standard deviation below the mean, respectively. As illustrated, traditionality significantly moderates the link between the SKHS and subordinates’ motivation for retaliation. When traditionality is low, SKHS has a considerable favorable effect on subordinates’ desire for retribution; when traditionality is high, it is fairly benign. High-traditional subordinates exhibit a reduced tendency for retaliation motivation compared to low-traditional subordinates when faced with the same level of SKHS.

To further examine the significance of the mediating effect of revenge motivation, we ran the PROCESS model 4 with 5,000 bootstrap samples. The indirect effect analysis results in Table 4 indicate that 0 is beyond the 95% confidence interval (LLCI = 0.007, ULCI = 0.098). Therefore, revenge motivation is a significant mediator in the link between SKHS and SKHC, which again confirms Hypothesis 4. In addition, we ran the PROCESS model 7 with 5,000 bootstrap samples to further examine the moderated mediating effect of revenge motivation. The index of moderated mediation in Table 4 shows that 0 is beyond the 95% confidence interval (LLCI = −0.067, ULCI = −0.002), which indicates significant moderated mediation effects. Therefore, revenge motivation plays a conditional mediating role in the relationship between SKHS and SKHC, which again confirms Hypothesis 5. Specifically, at −1 SD of traditionality, the mediation effect of revenge motivation in the relationship between SKHS and SKHC is significant (95% confidence interval = [0.012, 0.145]), whereas at +1 SD of traditionality, it is not (95% confidence interval = [−0.011, 0.069]).

**TABLE 4:** The results of mediation effect and moderated mediation effect.

Mediator	Effect	Boot SE	[LLCI, ULCI]
**Indirect effect of SKHS on SKHC**			
Revenge motivation	0.041	0.023	[0.007, 0.098]
**Index of moderated mediation**			
Revenge motivation	–0.024	0.016	[−0.067, −0.002]
**Conditional indirect effect(s) of SKHS on SKHC at values of the moderator (traditionality)**			
Revenge motivation at −1 SD of traditionality	0.063	0.034	[0.012, 0.145]
Revenge motivation at 1 SD of traditionality	0.016	0.019	[−0.011, 0.069]

## Discussion

Based on the displaced aggression theory, this paper constructs a theoretical model of supervisor malicious knowledge hiding from subordinates leading to malicious knowledge hiding of the subordinate from colleagues and discusses its mediation mechanism and boundary.

The results show that there is a trickle-down effect of malicious knowledge hiding, in which supervisor knowledge hiding has a significant positive impact on malicious knowledge hiding by subordinates to their colleagues. This study highlights the mediating role of revenge motivation between SKHS and SKHC. The findings also highlight the important role of personal traditionality in reducing the trickle-down effect of supervisor knowledge hiding. Traditionality can alter how individuals perceive and assess a supervisor’s mistreatment behavior, which in turn changes how they react to it. According to our findings, when confronted with a supervisor’s knowledge hiding, high traditionalist subordinates show a lower tendency of retaliation motivation than low traditionalist subordinates.

### Theoretical contributions

The theoretical contributions of this study are reflected in the following three aspects. First, scholars have conducted extensive research on knowledge hiding behavior in the horizontal direction, while little research has explored top-down knowledge hiding behavior (Arain et al., 2020c,^2022^; Butt, 2020), which may be more harmful to organizations than horizontal knowledge hiding (Connelly and Zweig, 2015). The effect of supervisor knowledge hiding on outcomes, such as attitudes, behaviors, and performance at the individual and organizational levels, is beginning to receive research attention. By focusing on the trickle-down effect and mechanism of the supervisor’s knowledge hiding behavior, this paper studies the influence chain of the destructive behavior of knowledge hiding within an organization from leaders to ordinary employees. It enriches the comprehension of the consequences of supervisor knowledge hiding (Offergelt and Venz, 2022) and adds to the research on trickle-down effect of negative aspects of leadership in organizations (Mawritz et al., 2012).

Second, researchers have examined the impact of knowledge hiding behavior from a variety of angles, including the theories of resource conservation, regulatory focus, territorial conduct, social exchange, and moral disengagement (Connelly et al., 2019; Afshan et al., 2022). Previous studies generally regarded knowledge hiding as a deviant behavior, and its deliberate aggressiveness nature is overlooked, because the rationalized hiding dimension of knowledge hiding is distinctly diverse from other dimensions and its harmfulness is controversial. This study focused on the malice, deceit, and harmfulness inherent in a superior’s knowledge hiding behavior toward subordinates. Playing dumb and evasive hiding, two obviously harmful aspects of knowledge hiding, were examined in this study. Based on the cognitive process model of “frustration-aggression” and the displaced aggression theory, this research explores the crucial role of retaliation motivation in inducing the hostile knowledge hiding conduct targeted at colleagues by subordinates. This study adds to previous research on the role of revenge motivation in the relationship between supervisor misbehavior and employee deviance (Skarlicki and Folger, 1997; Mitchell and Ambrose, 2007; Hung et al., 2009). This study also advances our understanding of the displaced aggression mechanisms that underlie the adversarial reaction of followers to the leaders’ mistreatment (Hoobler and Brass, 2006; Restubog et al., 2011).

Third, situational factors must be taken into account in order to fully understand how the attitudes and behaviors of leaders affect their subordinates. This is especially true in societies where there is a strong respect for authority and a high degree of power distance, in which the effects of negative leader behavior are often quite different. The study of contextual variables that may lessen or even prevent the trickle-down of supervisors’ knowledge-hiding to subordinates is a fascinating and crucial topic that merits further investigation (Arain et al., 2022; Jasimuddin and Saci, 2022; Offergelt and Venz, 2022). Traditionality is a variant on power-distance, but at an individual level for analysis (Farh et al., 1997). Traditionality deeply reflect the value orientation of individuals, which are likely to vary among individuals within a nation and are particularly pertinent to the hierarchy inherent in leadership (Spreitzer et al., 2005). Its role in the organization has been tested not only in the eastern context but also in the western context (Spreitzer et al., 2005). This paper introduces traditionality into the recently emerging research field of supervisor malicious knowledge hiding, which not only enhances research on the function of traditionality in mitigating the relationship between interpersonal mistreatment and employee deviance (Farh et al., 2007; Liu et al., 2010; Zhao et al., 2013) but also advances knowledge of the conditional mechanisms underlying the influence of supervisor malicious knowledge hiding behavior (Arain et al., 2020a,b).

### Managerial implications

This study finds that a supervisor’s malicious knowledge hiding from subordinates will lead subordinates to engage in covert forms of retaliation. As a result, it is critical to gain insight into the driving forces behind the supervisor’s knowledge hiding behavior and prevent such misbehavior at the source. Companies should put in place preventive measures to improve the rapport between leaders and employees and ease leaders’ concerns about knowledge and information exchange. Enterprises should also enforce strict consequences for leaders with poor management styles, create a transparent and effective feedback system for subordinates, and protect subordinates from a supervisor’s vicious reprisals.

This study also reveals that, by inciting subordinates’ revenge motivation, a supervisor’s knowledge hiding activity will cause them to engage in retaliatory negative coping behaviors. Therefore, it is necessary to disable its intermediary mechanism to prevent the harmful effects caused by the supervisor’s malicious knowledge hiding conduct. Enterprises should set up systems to quickly and effectively identify and manage the negative feelings of employees in response to the frustration and rage created by supervisors hiding knowledge. In addition, an important source of revenge motivation is that employees’ resource needs are not addressed, and their work autonomy, competence, and sense of belonging to the organization are compromised. To reduce the circumstance where the resource demand cannot be met due to supervisor knowledge hiding, companies should create multi-level and multi-dimensional support systems to provide employees with sufficient support for learning improvement and career advancement.

This study also confirms the role of the traditionality of subordinates in moderating the impact of supervisor knowledge hiding on a subordinate’s desire for retribution. This finding provides a crucial foundation for businesses looking to control the negative influence of a leader’s negative behavior. Subordinates with less traditionality place more importance on the reciprocal exchange-based relationship balance, where supervisor knowledge hiding is more likely to trigger their revenge motivation. To reduce the desire for retaliation and harmful reciprocity, enterprises can evaluate the traditionality of employees in some daily human resource management activities, such as performance communication and team building activities, and pay closer attention to low-traditionality employees, offering them timely psychological counseling and resource demand support to help them integrate into the firm and adjust to a particular working environment.

### Limitations and directions for future research

This study confirms the effect of supervisor knowledge hiding behavior on the poor coping behavior of subordinates from the standpoint of displaced aggression. Despite the theoretical viewpoint being novel, there is still room for further discussion and development. First of all, displaced aggression encompasses both the replacement of the behavior mode and the behavior object. This study focuses on behavior object substitution, in which the subordinate transfers the hostility brought on by the supervisor to other employees. However, subordinate substitution attacks take many different forms, one of which is knowledge hiding behavior toward colleagues. Future studies can enrich the understanding of whether subordinates also engage in less covert and more detrimental negative behaviors, such as workplace aggression, deviant behavior, and counterproductive activity.

In addition, this paper mainly focuses on the cognitive process of “frustration-aggression.” However, an individual’s emotions play an important role. Berkowitz (1989) identified the emotional mediator in the “frustration-aggression” model, and Liu et al. (2015) confirmed the critical role of emotional exhaustion in the triggering of alternative aggression by external conflict based on the theory of displaced aggression. Therefore, to further connect the supervisor’s malicious knowledge hiding and the unfavorable coping mechanisms of subordinates, future research should combine emotional mechanisms to explore the direct and indirect effects brought on by the supervisor’s malicious knowledge hiding actions.

Third, this paper considers the malicious and invasive characteristics of supervisor knowledge hiding behavior and focuses on the two dimensions of playing dumb and evasive hiding. However, like most of the existing literature on knowledge hiding, we measure knowledge hiding as a whole rather than distinguish specific dimensions. Playing dumb and evasive hiding, on the other hand, may have various effects on subordinates’ unfavorable reactions due to variances in the degree of covertness, deceit, malice, and prevalence. Therefore, in order to better understand this phenomenon and aid in the development of work policies, we propose that the various elements of supervisor knowledge hiding behavior are studied separately in future work.

Furthermore, drawing from Wo et al. (2015) highlighted “multiple mediation processes” of trickle-down effect, a fascinating extension of this research would be to develop and examine a model that incorporates various theories of trickle-down effects for SKHC, such as social exchange theory and social learning theory, and identify and measure mediating variables representing each theory. It will improve our comprehension of the mediating processes of SKHC trickle-down effect as well as the practical implications for those interested in managing it within enterprises.

## Conclusion

This paper explored the effect of supervisor knowledge hiding on subordinate knowledge hiding. We focused on the malicious and invasive characteristics of knowledge hiding, introduced the theory of displaced aggression and the cognitive process of “frustration-aggression,” and examined the intermediate mechanism by which a supervisor’s knowledge hiding from subordinates affects the subordinate’s knowledge hiding from colleagues. The research confirmed that supervisor knowledge hiding was aggressive behavior that could arouse employees’ desire for retaliation, which in turn could lead to employees’ knowledge hiding behaviors to other colleagues. The generation of such revenge motives varied among employees with different traditionality characteristics. This research demonstrates the trickle-down effect of supervisor knowledge hiding behavior, enhances top-down knowledge hiding research on its effects and influencing mechanisms, and provides further insight into organizational practice.

## Data availability statement

The raw data supporting the conclusions of this article will be made available by the authors, without undue reservation.

## Ethics statement

Ethical review and approval was not required for the study on human participants in accordance with the local legislation and institutional requirements. The patients/participants provided their written informed consent to participate in this study.

## Author contributions

YT was in charge of the research design and guidance. H-MZ was in charge of the research implementation, data analysis, and manuscript writing. XD was in charge of the questionnaire survey, data analysis, and draft of manuscript. All authors read and approved the final version of the manuscript.
